# Intercolony variation in foraging flight characteristics of black‐headed gulls *Chroicocephalus ridibundus* during the incubation period

**DOI:** 10.1002/ece3.6291

**Published:** 2020-06-01

**Authors:** Dariusz Jakubas, Piotr Indykiewicz, Jarosław Kowalski, Tomasz Iciek, Piotr Minias

**Affiliations:** ^1^ Department of Vertebrate Ecology and Zoology Faculty of Biology University of Gdańsk Gdańsk Poland; ^2^ Department of Biology and Animal Environment Faculty of Animal Breeding and Biology UTP University of Science and Technology Bydgoszcz Poland; ^3^ Sierpc Poland; ^4^ Łódź Poland; ^5^ Department of Biodiversity Studies and Bioeducation Faculty of Biology and Environmental Protection University of Łódź Łódź Poland

**Keywords:** foraging ecology, GPS tracking, gulls, incubation

## Abstract

Using GPS loggers, we examined the influence of colony, sex, and bird identity on foraging flight characteristics of black‐headed gulls *Chroicocephalus ridibundus* during the incubation period. We studied tracks of 36 individuals breeding in one urban and two rural colonies in Poland. Birds from both rural colonies performed the furthest flights (mean max distance 8–12 km, up to 27 km) foraging mainly in agricultural areas. Gulls from the urban colony performed shorter flights (mean 5 km, up to 17 km) visiting mainly urbanized areas and water bodies. We found that females performed longer flights and their flight parameters were less repeatable compared to males. Males from both rural colonies visited water bodies more frequently than females. In all colonies, males (but not females) used habitats unproportionally to their availability in the vicinity. Relatively low interindividual and relatively high intraindividual overlap in home ranges indicated considerable foraging site fidelity. Individuals specialized in the use of a particular type of habitat performed shorter foraging flights compared to individuals using diverse habitats during their foraging flights. Our results indicate diverse foraging strategies of black‐headed gulls, including generalists that explore various habitats and specialists characterized by high foraging site and habitat fidelity.

## Graphical abstract

Birds from both rural colonies performed the furthest flights (mean max distance 8‐12 km, up to 27 km) visiting mainly agricultural areas; gulls from urban colony performed shorter flights (mean 5 km, up to 17 km) visiting mainly urbanized areas and water bodies. We found that females performed longer flights and their flights parameters were less repeatable compared to males. Our results indicate diverse foraging strategies of black‐headed gulls, including generalists that explore various habitats and specialists characterized by high foraging site and habitat fidelity.

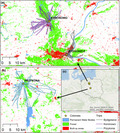

## 1 INTRODUCTION

Spatial movements of birds during the breeding period provide important insights into food availability and foraging ecology. Characteristics of foraging flights (range, duration) often vary considerably at both inter‐ and intraspecific levels. This variability is driven by a multitude of factors including local environmental conditions, within‐ and between‐species competition, individual experience, age, sex, reproductive status, and phase of the annual cycle (e.g., Estévanez & Aparicio, 2019; González‐Solís, Phillips, Daunt, Lewis, & Wilson, 2017; Kazama et al., 2018; Noordhuis & Spaans, 1992).

Foraging strategies may be site‐specific, that is, shaped by local conditions including food distribution, distance to the food patches, local topography, or climate conditions. Colonially breeding birds are central‐place foragers, which exploit food sources located directly around their colonies. Local food availability and distribution, as well as the need to optimize the time and energy expenditures on travel to foraging grounds and food acquisition, are thought to determine the predominant foraging strategies in particular colonies. As a consequence, birds from closely located colonies may adopt various feeding strategies and be partially or completely spatially segregated (e.g., Bolton, Conolly, Carroll, Wakefield, & Caldow, 2019; Masello et al., 2010; Wanless & Harris, 2006). This segregation in space and variation in foraging strategies may result from avoidance of competition, high foraging site fidelity, and colony‐wide information transfer where individuals follow the successful foragers (Barta, 2001; Richner & Heeb, 1995).

Sexual differences in foraging strategies have been reported for many avian species and may be a consequence of habitat specialization and/or sex‐specific nutrient requirements (e.g., Blount, Houston, Surai, & Møller, 2004; Hedd, Montevecchi, Phillips, Fifield, & Garthe, 2014; Phillips, McGill, Dawson, & Bearhop, 2011; Phillips, Silk, Phalan, Catry, & Croxall, 2004) or serve as a mechanism reducing intersexual competition. Differences in foraging behavior between males and females in sexually dimorphic species have often been attributed to morphological differentiation, which can affect foraging efficiency or competitive ability. Partial or complete sexual foraging segregation has been reported for several sexually dimorphic and monomorphic species, including gulls (e.g., Camphuysen, Shamoun‐Baranes, Loon, & Bouten, 2015; Cleasby et al., 2015; Ismar, Raubenheimer, Bury, Millar, & Hauber, 2017).

Many animals, independently of their sex, age, breeding phase, and reproductive status, show feeding specializations that may be expressed in individual‐specific diet composition, fidelity to feeding sites, consistency in foraging trip characteristics, or habitat use in the short‐ or long‐term scale (Bolnick & Kirkpatrick, 2012; Dall, Bell, Bolnick, & Ratnieks, 2012; Phillips, Lewis, González‐Solís, & Daunt, 2017). Since foraging specialization may reduce niche overlap and resource competition among individuals, its adaptive value is especially evident in high‐competition environments (Bolnick, 2001; Svanbäck & Bolnick, 2007).

Gulls (*Laridae*) are a good avian model group to study variability of foraging strategies and foraging ecology. Their opportunistic foraging enables them to feed on various food sources in various habitats by adopting individually distinct foraging strategies. Thus, they are referred to as an example of a generalist population consisting of specialist individuals (e.g., Navarro et al., 2017; Ramos, Ramírez, Sanpera, Jover, & Ruiz, 2009).

**FIGURE 1:**
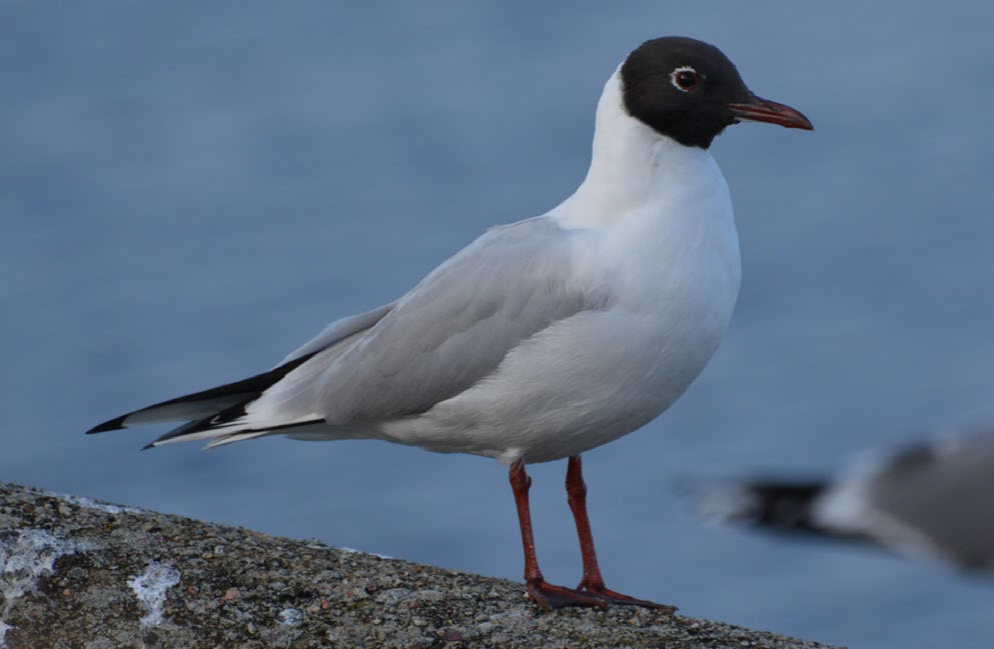
An adult black‐headed gull *Chroicocephalus ridibundus* in the breeding plumage

In this study, we used GPS tracking to describe movement patterns and habitat use of the black‐headed gull *Chroicocephalus ridibundus* (hereafter BHG) during the incubation period. This waterbird (Figure 1) is characterized by colonial breeding and sexual size dimorphism (Indykiewicz, Minias, Kowalski, & Podlaszczuk, 2019; Palomares, Arroyo, Marchamalo, Sainz, & Voslamber, 1997). The diet of this omnivorous gull consists of invertebrates (especially earthworms), plants (seeds, agricultural grain), fish, and anthropogenic food waste in urban areas (Cuendet, 1983; Gotmark, 1984; Kubetzki & Garthe, 2003; Vernon, 1972a). Despite relatively numerous studies on BHG foraging ecology (e.g., Andersson, Götmark, & Wiklund, 1981; Brandl & Nelsen, 1988; Cuendet, 1983; Gotmark, 1984; Moreira, 2007; Schwemmer & Garthe, 2008; Vernon, 1972a), to our knowledge, radiotelemetric studies of foraging flights have been conducted so far only in Bavaria, Germany (Brandl & Gorke, 2007; Gorke & Brandl, 1986). Hence, basic knowledge on their individual spatial behavior is missing. One may expect that similarly to other gulls, BHGs will show at least some degree of habitat or prey specialization expressed as individual foraging site fidelity (Camphuysen et al., 2015; Enners, Schwemmer, Corman, Voigt, & Garthe, 2018; Navarro et al., 2017; Van Donk, Shamoun‐Baranes, Bouten, Meer, & Camphuysen, 2019). To test this general hypothesis, we examined variation in the foraging ecology of BHGs from three colonies in Central and Northern Poland.

First, we aimed to determine if foraging trip characteristics (maximal range, total distance covered, total duration, and frequency of visits in particular habitat types) and habitat use differed between the colonies. We expected that distances covered by BHGs from particular colonies and habitat use during foraging trips should differ between the colonies due to preferences of some optimal habitats types; that is, we expected that birds may select particular types of foraging habitats unproportionally to their availability around particular colonies. Specifically, we expected a more frequent use of urbanized areas by birds from an urban colony.

Second, we investigated sex differences in foraging trip characteristics and habitat use. We expected that due to sexual dimorphism in morphology, especially in head length, bill length, and bill depth (Indykiewicz et al., 2019; Palomares et al., 1997), foraging flight characteristics of BHGs should differ between sexes—for example, larger males may explore more demanding habitats with a higher number of competitors. Sexual dimorphism may result in competitive interference within feeding habitats, with smaller females being displaced more frequently than males and, hence, being less site‐faithful. Indeed, previous studies on larger gull species reported sex differences in foraging and/or diet to competition level over food resources in particular habitats (e.g., Camphuysen et al., 2015; Greig, Coulson, & Monaghan, 1985; Navarro et al., 2010). Thus, we also expected differences in habitat use and/or selectivity between sexes. In particular, we expected lower repeatability of foraging flight characteristics and site fidelity in females.

Third, we studied individual components of foraging flight characteristics, site fidelity, and habitat selection. While dietary studies have presented many gulls as generalists at the population level, telemetry data often revealed strong behavioral and habitat specialization at the individual level (e.g., Juvaste et al., 2017; Maynard & Ronconi, 2018; Navarro et al., 2017; Ramos et al., 2009). Thus, we expected low interindividual similarities in foraging flight characteristics at the population level and clear spatial segregation of core areas. We also expected high intraindividual repeatability in flight characteristics and considerable foraging habitat and spatial specialization of particular individuals.

Finally, we investigated whether habitat use affected flight characteristics. We expected that specialized individuals exploiting only a specific habitat type may be more efficient in foraging and spend less time on feeding compared to generalists foraging across a wider spectrum of habitats.

## 2 MATERIALS AND METHODS

### 2.1 Study area

**FIGURE 2:**
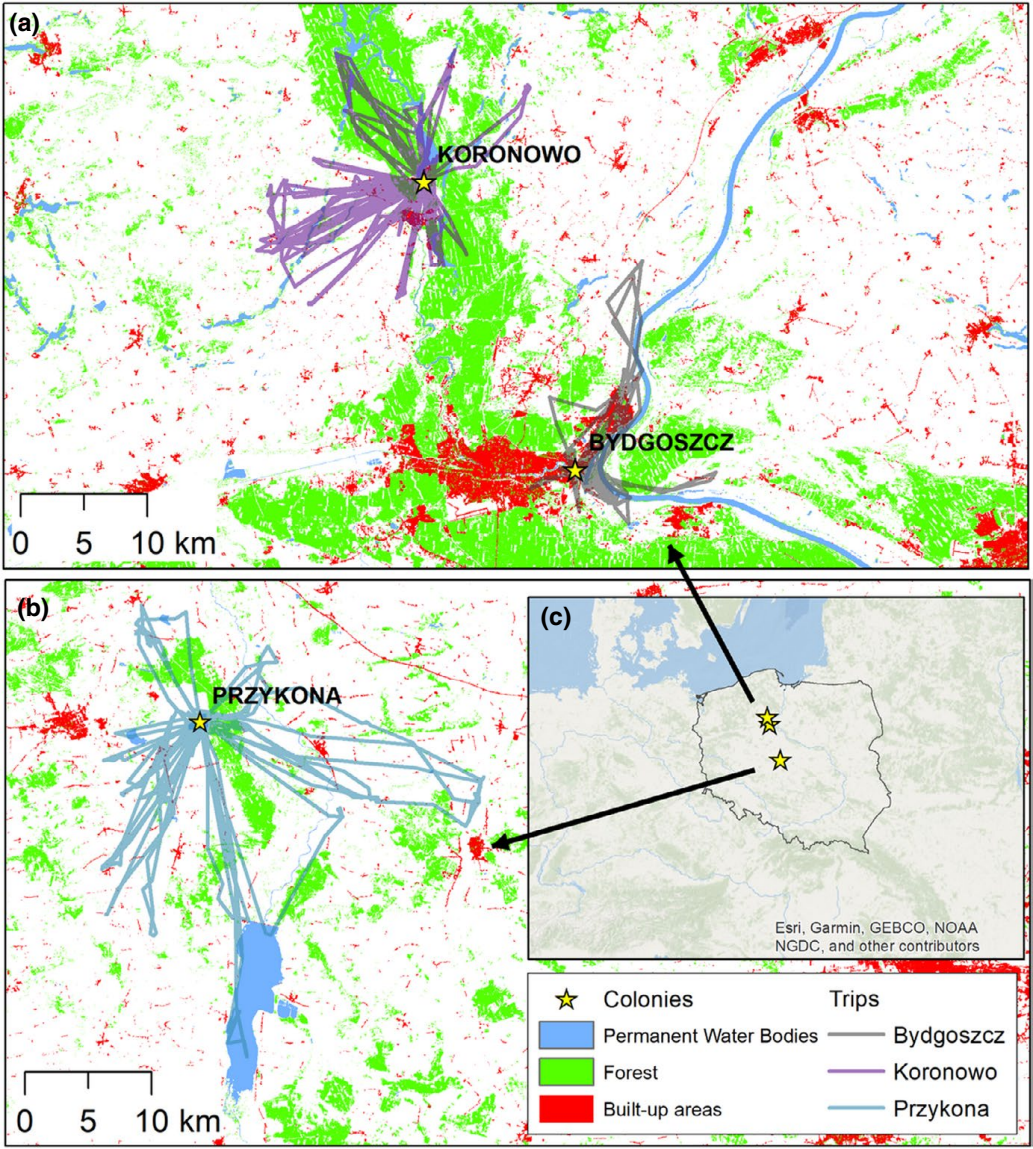
All foraging flights of GPS‐tracked black‐headed gulls breeding in three colonies in N Poland. Maps created based on data from Pan‐European High Resolution Layer: Imperviousness Degree (IMD) 2012, Forest Type (FTY) 2012, and Permanent Water Bodies (PWB) 2012 (European Environment Agency (EEA); https://land.copernicus.eu/pan‐european/high‐resolution‐layers)

BHGs were captured in 2018 in three colonies located across Central and Northern Poland (located in the North European Plain). All study colonies differed in the habitat structure around the breeding locations (Figure 2). The first colony (320 breeding pairs) was situated in an urban zone of Bydgoszcz City (53°7ʹ7.9ʺN 18°6ʹ19.3ʺE; 350,000 inhabitants) on a small islet at the eutrophic Brda River, which offered high availability of food in close vicinity of the colony. The area around the colony was highly urbanized with factories, service buildings, and a railway line located nearby. The second colony (660 breeding pairs) was situated on a small islet (53°20′3.8ʺN, 17°57′53.7ʺE) in the northern part of Koronowskie Lake (1,560 ha), an artificial reservoir established in the late 1960s. The lake is used for recreation, and seasonal holiday houses are located near the island. It is situated 0.1 km from the dam of Koronowskie Lake surrounded by green areas, and 2.5 km from the center of the Koronowo town (12,000 inhabitants). Finally, the third colony (ca. 3,000 breeding pairs) was located on a 3.5 ha islet (52°00ʹ20.6ʺN 18°39ʹ28.2ʺE) at an artificial Przykona reservoir (165 ha) established after lignite exploitation in 2004. This was a mixed gull colony with a small number of gulls from other species, including Caspian gulls *Larus cachinnans* (<10 pairs) and Mediterranean gulls *Ichthyaetus melanocephalus* (<10 pairs), breeding there. The reservoir was located 9 km from the medium‐size town of Turek (27,000 inhabitants). The three study colonies are henceforth referred to as Bydgoszcz, Koronowo, and Przykona, respectively.

### 2.2 Habitat structure around the colonies

Habitat structure in the 27‐km radius around each colony (this distance is equal to maximal flight distance from the colony recorded in this study) differed significantly between the colonies (Table 1). The vicinity of the Przykona colony was characterized by the significantly larger share of agricultural areas compared to other colonies. Areas around the Bydgoszcz colony were characterized by the significantly lower contribution of agricultural areas compared to Koronowo. At the same time, the area of artificial surfaces and forests around the Bydgoszcz colony was significantly larger than in other colonies (Table 1). The area of water bodies in the buffers around all colonies was similar (Table 1). Considering these differences in habitat structure in the 27‐km buffers and the proportion of artificial areas in a direct vicinity (within a 2‐km radius) of each colony (i.e., 51.9% at Bydgoszcz, 6.9% in Koronowo and 4.0% at Przykona), the colony at Bydgoszcz is henceforth referred to as urban, while the other two colonies are referred to as rural.

**TABLE 1:** Habitat structure, that is, relative abundance [%] of particular habitat types (according to CORINE land cover CLC2018 model, level 1) in buffers within a 27‐km radius from particular black‐headed gull colonies (this distance is equal to maximal distance from the colony recorded in this study); *χ* ^2^, *p*—results of intercolony comparison of particular habitat types (*χ* ^2^ tests of independence performed on raw data in km^2^, *df* = 2)

Habitat type (CLC2018, level 1)	Area [%]			Difference	
	Bydgoszcz	Koronowo	Przykona	*χ* ^2^	*p*
Agricultural areas	52.9^a,b^	62.1^a,c^	73.1^b,c^	45.82	<.001
Artificial surfaces	8.5^a,b,c^, ^d,e^	6.9^d,e^	4.8^a,b,c^, ^d,e^	21.24	<.001
Forest and seminatural areas	36.9^a,b^	28.4^a,c^	20.1^b,c^	88.69	<.001
Water bodies	1.7	2.4	1.7	4.26	.119
Wetlands	0.1	0.2	0.3	—	—

Note

Data taken from CORINE land cover model CLC2018 (https://land.copernicus.eu/pan‐european/corine‐land‐cover/clc2018).

^a,b,c^ Significant differences (*p* < .004).

^d,e^ Tendency to difference (*p* < .06) in proportions of particular habitat types tested on raw data by 2 × 2 *χ* ^2^ tests of independence (*df* = 1). Wetlands were not analyzed due to the small area in each buffer (<0.5% of the total buffer area).

### 2.3 GPS tracking

To characterize flights of BHGs during the incubation period, we used 18 PinPoint‐10 GPS store‐on‐board loggers (1.3 g; Lotek Wireless Inc.) recording time and position. In total, we deployed loggers on one pair member from 37 nests (10 at Bydgoszcz, 16 at Koronowo and 11 at Przykona). We captured birds on nests during the 2nd–4th week of the incubation period using spring traps (ECOTONE) or loops made of nylon lines. The tags were attached to the central back feathers of each bird using two crosswise‐applied, 3‐ to 4‐mm‐wide strips of Tesa tape code 4,965 (Tesa Tape Inc.) at approximately the midpoint of the centerline of the body. The tag weight (including attachment = 1.6 g) was equivalent to an average of 0.75% and 0.64% body mass of the captured females and males, respectively (data for birds from all colonies combined). We acknowledged that behavior, time, and energy budget of birds may be directly affected by externally attached devices via (1) expending extra energy countering both the additional mass and the increased drag and (2) decreasing some aspects of their performance, such as speed (Elliott, Davoren, & Gaston, 2007; Vandenabeele, Shepard, Grogan, & Wilson, 2012). This bias might have affected the pattern of foraging trips observed in the present study. Nevertheless, as birds from all studied colonies were equipped with the same type of GPS loggers, we assume that any possible bias was similar in all studied sites.

Loggers were programmed to collect data at 15‐min intervals and were used during 10–26 May at Bydgoszcz, 28 April–23 May at Koronowo, and 19–31 May at Przykona. After about 48 hr from logger deployment, we tried to recapture birds and retrieve tags. We successfully recaptured 36 (97.3%) marked birds; a single individual from Koronowo abandoned the nest and could not be recaptured. We successfully downloaded data from all tags, but we excluded data from one individual from Koronowo due to the wrong interval setting. In total, we analyzed 129 trips from 35 individuals, including 16 females and 19 males. All birds were molecularly sexed using a drop of blood collected from the ulnar vein upon bird recapture and tag retrieval. DNA was extracted using a Genomic DNA Purification Kit (Thermo Fisher Scientific) according to the kit protocol. Molecular sexing was based on the analysis of the sex‐linked chromo‐helicase DNA‐binding (CHD) gene which we amplified using the primers and protocol developed by Griffiths, Double, Orr, and Dawson (1998).

### 2.4 Statistical analyses

Based on the geographical positions recorded by the GPS loggers, we analyzed the following foraging flight characteristics: (a) maximum range of flights—distance (km) from the colony to the distal point reached on each foraging trip; (b) the total distance covered (km) as the sum of the distances (km) between all GPS positions along each individual's track; and (c) total trip duration, defined as the time between departure and return to the colony (min).

A foraging trip was defined as a trip with at least three positions recorded with a minimum distance of 1 km from the colony. We analyzed only flights performed during day hours (4:00–23:00), as some individuals from the Bydgoszcz colony were regularly spending nights on the roofs of buildings in the city center and returning to the colony during the daylight hours.

To analyze foraging flight characteristics, we used separate linear mixed models (LMM) with maximum range of flights, the total distance covered and total trip duration as response variables, colony, sex, and interaction between them as predictors, and bird identity as a random factor. We used post hoc estimated marginal means test. To estimate the significance of the random effect in LMM analyses, we compared models with and without random effect using *F* test with the Kenward–Roger approximation (Halekoh & Højsgaard, 2014). We performed LMM analyses in the *lmer*, *lme4*, and *pbkrtest* packages (Halekoh & Højsgaard, 2014) and post hoc estimated marginal means test in the *emmeans* package in R (R Core Team, 2018).

We assessed whether the data sufficiently met the assumptions of the linear model using *Q*–*Q* plots (quantile expected in normal distribution versus quantile observed plot for residuals). As the distribution of the obtained data was not normal, we log‐transformed the analyzed flight parameters, resulting in residuals with the normal distribution. To investigate relationships of the total trip duration with total distance covered and maximal distance covered, we used the Pearson product–moment correlation coefficient.

In order to examine consistency in foraging flight characteristics, we calculated repeatability *R* in maximum range of flights, total distance traveled, and total trip duration using the *rptR* package in R (Stoffel, Nakagawa, & Schielzeth, 2017).

We quantified habitat and spatial specialization based on GPS fixes classified as “foraging” only. To select these GPS positions, we used an expectation‐maximization binary clustering for behavioral annotation. This technique uses velocity and turning angle obtained from successive locations to cluster GPS positions in four behavioral categories (Garriga, Palmer, Oltra, & Bartumeus, 2016): high velocity/low turn (HL), high velocity/high turn (HH), low velocity/low turn (LL), and low velocity/high turn (LH). We considered as foraging position all positions recorded >1 km from the colony characterized by low velocity/low turn (LL) or low velocity/high turn (LH) and representing most probably walking, looking for food, standing while looking for food, handling prey, other small‐scale movements. We applied the clustering algorithm for particular studied individuals using the R package *EMbC* (Garriga et al., 2016).

To visualize core areas exploited by birds from all colonies, we calculated utilization distributions (UDs, probability distributions constructed from data providing the location of an individual in space at different points in time) for home ranges (95% kernel density) and core ranges (50% kernel density) for foraging position in Geospatial Modelling Environment (GME) ver. 0.7.4.0 software (www.spatialecology.com/gme/) using the kde function and plugin bandwidth selection.

To investigate spatial similarity of flights, we calculated intra‐ and interindividual overlaps of home ranges using the *adehabitatHR* package (Calenge, 2006) in R (R Core Team, 2018) with algorithm BA, that is, Bhattacharyya's affinity, a statistical measure of affinity between two populations. It ranges from zero (no overlap) to one (identical UDs; Calenge, 2006). In these analyses, we considered only trips with at least five GPS positions recorded.

To quantify foraging habitat use and specialization, we determined habitat types in foraging positions based on the level 1 data from the CORINE Land Cover (CLC) 2018, version 20b2. CLC uses a minimum mapping unit of 25 ha and land cover classes are grouped in a three‐level hierarchy with ascending number of land cover classes from 5 to 44 (https://land.copernicus.eu/pan‐european/corine‐land‐cover/clc2018); due to relatively small sample size, we used level 1 with five types of land cover distinguished.

We compared the proportion of GPS foraging locations recorded in different habitats firstly between birds from different colonies and secondly between males and females from each colony, using chi‐square or Fisher's exact test using the MASS package in R (R Core Team, 2018). To examine whether birds forage opportunistically across different available habitats or are specialized in exploitation of limited habitat types, we compared the proportion of foraging positions recorded in each habitat to the proportion of habitat types (CORINE CLC 2018 level 1 land cover) available within the observed flight range (27 km). Habitat selection of BHGs was computed using Manly's compositional analysis of resource (habitat) selection ratios for II data type (the same habitat availability for all animals, but habitat use measured for each one) combined with 95% Bonferroni simultaneous confidence intervals (Manly, McDonald, Thomas, McDonald, & Erickson, 2007). The Manly selection ratios from 0 to 1 indicate habitat types used less than available or avoided, whereas selection ratios >1 indicate habitats used more than available or preferred (Ramesh, Kalle, & Downs, 2016). We considered significant habitat preference if the lower *CI* limit was above 1 and avoidance if the upper *CI* limit was below 1 (Manly et al., 2007). To test if all individuals used habitats in the same proportions (irrespective of the habitat selectivity), we used a log‐likelihood chi‐square test (Khi2L1). To test overall habitat selection, that is, use in proportion to their availability, we used Khi2L2 test. To test the hypothesis that animals are on average using resources in proportion to availability, irrespective of whether they are the same or not, we used Khi2L2–Khi2L1 test (Manly et al., 2007). We performed habitat selection analyses for all individuals and then separately for males and females. We used the *adehabitatHS* package (Calenge, 2011) in R software to perform resource selection analyses according to Manly et al. (2007) using a log‐likelihood nonrandom statistic.

To test whether habitat specialization may affect flight parameters, we calculated diversity of habitat use (DHU) based on Shannon index:

$$\text{DHU}=-\frac{\sum p_{i}\ln\left(p_{i}\right)}{\ln(3)}$$

where *pi* is the proportion of agricultural areas, artificial surfaces, or water bodies in foraging positions visited by the GPS‐tracked individuals during the incubation period. This metric ranges from 0 to 1, where a higher value implies a higher consistency in foraging habitat use. We considered only individuals with at least two foraging positions. In total, we analyzed 74 positions of 34 individuals. Then, we studied relationships between DHU and foraging flight duration and total distance covered. As many individuals have DHU = 1 resulting in non‐normal data distribution, we used Kendall–Theil Sen–Siegel nonparametric linear regression using the *mblm* package in R (Mangiafico, 2016) to investigate relationships of DHU with total flight duration and total distance covered.

To estimate the proportion of individuals specialized in foraging in a particular type of habitat, we divided particular flights (only those with at least two foraging positions recorded) and individuals based on habitat use in the foraging positions into two categories: (1) with predominant relative ratio (>95%) of foraging position in one type of habitat and (2) with nonpredominant relative ratio (≤95%) of foraging position in one type of habitat. Then, we calculated the proportion of flights and individuals using predominantly a single habitat type. Finally, we compared total flight duration and total distance covered between the flights characterized by two different categories of habitat use using the Wilcoxon test.

In all statistical analyses, we set the alpha level at 0.05. We mapped data from the GPS loggers, extracted data from the CORINE model and produced all figures with maps using ArcMap/ArcGIS 10.3.1 (Environmental Systems Research Institute). All values are reported as means ± *SD*.

## 3 RESULTS

### 3.1 Variation in foraging flight characteristics

During the incubation period, black‐headed gulls (BHG) from the studied colonies performed flights lasting 71–732 min (mean±SD 172.1 ± 126.6 min) with the maximal range of flights ranging from 1 to 27 km (8.2 ± 5.5 km). The total distance of a single foraging flight ranged from 2 to 62 km (19.0 ± 13.4 km; *n* = 96 flights of 35 individuals; Figure 2). There was significant variation in all foraging flight characteristics between the colonies and the random effect (bird identity) was significant in each model (Table 2).

**TABLE 2:** Linear mixed‐effects models estimating the effects of colony, sex, their interaction, and bird identity on foraging flight parameters

Variable	Maximal flight range			Total distance covered			Log total flight duration		
	*df*	*F*	*p*	*df*	*F*	*p*	*df*	*F*	*p*
Colony	2	10.16	**.001**	2	11.83	**.000**	2	11.63	**.000**
Sex	1	0.16	.693	1	0.32	.574	1	5.68	**.019**
Colony × Sex	2	0.06	.944	2	0.02	.981	2	2.78	0.067
r.e. Bird_ID	5	4.61	**.003**	5	5.22	**.002**	5	7.19	**.000**

Note

Significance of random effect (bird identity) estimated by *F* test with the Kenward–Roger approximation. Significant effects are bolded.

Birds from the rural colony at Przykona performed significantly further and longer foraging flights than birds from the other two colonies, as measured with maximal flight range (11.8 ± 6.7 km, range: 1–27 km), total flight distance (28.7 ± 17.5 km, range: 2–62 km), and total flight duration (265 ± 194 min, range: 71–732 min; all pairwise comparisons: *p* < .030). There were no significant differences in foraging flight characteristics between birds from Koronowo and Bydgoszcz (all pairwise comparisons: *p* > .05), although maximal flight range tended to be shorter in the latter colony (8.5 ± 3.5 km, range: 2–14 km in Koronowo vs. 4.6 ± 4.2 km, range: 1–17 km in Bydgoszcz; *p* = .086). In contrast to the maximal and total distances of foraging flights, the total trip duration was also affected by sex (Table 2) with females from all colonies performing flights of significantly longer duration (210 ± 151 min, range: 71–732 min) compared to males (143 ± 95 min, range: 72–715 min).

**FIGURE 3:**
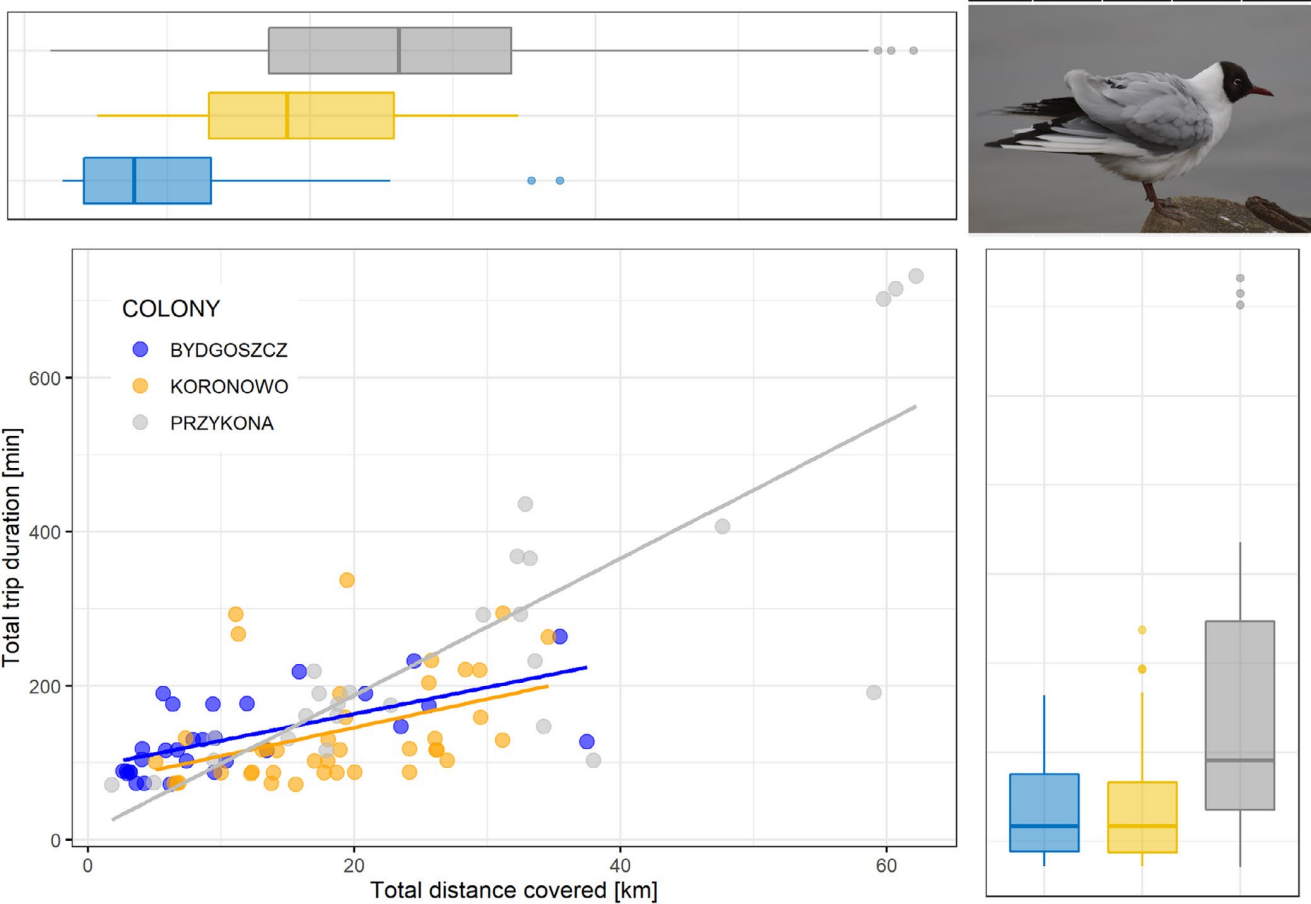
Distribution of total distance covered [km] and total trip duration [min] of flights performed by GPS logger‐equipped black‐headed gulls from three breeding colonies during the incubation period. The top boxplot shows total distance covered, and the right one, total trip duration. Boxplots show the median (band inside the box), the first (25%) and third (75%) quartile (box), the lowest and the highest values within 1.5 interquartile range (whiskers) and outliers (dots). Least squares regression lines are shown for significant relationships. Photograph by Dariusz Jakubas

We found a positive significant relationship between the total distance covered and the total trip duration (log‐transformed) of BHGs breeding at Bydgoszcz (*r* = .64, *df* = 28, *p* < .001), Koronowo (*r* = .48, *df* = 38, *p* = .002), and Przykona (*r* = .80, *df* = 24, *p* < .001; Figure 3). A significant positive relationship was also found between the maximal flight range and total trip duration (log‐transformed) in each colony (Bydgoszcz: *r* = .55, *df* = 28, *p* = .002; Koronowo: *r* = .38, *df* = 38, *p* = .017; Przykona: *r* = .67, *df* = 24, *p* < .001).

### 3.2 Repeatability of foraging flight characteristics

We found that BHGs from the colonies at Bydgoszcz and Przykona were significantly repeatable in their maximal flight ranges, while birds from Koronowo showed a marginally nonsignificant tendency for repeatability of this trait (Table 3). Total flight distance and duration showed no significant repeatability (Table 3). However, sex‐specific analysis showed that only males were significantly repeatable in their maximal flight ranges across all the colonies. Males also showed significant repeatability in total distance covered at Koronowo and Przykona (Table 3).

**TABLE 3:** Repeatability of foraging flight characteristics (maximal flight range, total distance covered, and total trip duration) for males and females and both sexes combined of black‐headed gulls breeding in particular colonies

Variables	Bydgoszcz			Koronowo			Przykona		
	Both sexes	Females	Males	Both sexes	Females	Males	Both sexes	Females	Males
*n* flights	30	11	19	40	18	22	26	13	13
*n* ind.	10	3	7	14	7	7	11	6	5
Maximal flight range									
*R*	0.274	0.306	0.436	0.288	0.217	0.453	0.454	0.000	0.881
*SE*	0.169	0.285	0.224	0.177	0.226	0.228	0.216	0.206	0.137
CI	0–0.6	0–0.814	0–0.731	0–0.605	0–0.634	0–0.742	0–0.748	0–0.661	0.481–0.971
*p*	**.049**	.237	.059	.065	.285	**.027**	**.034**	1.000	**.001**
Total distance covered									
*R*	0.205	0.248	0.295	0.175	0.037	0.461	0.201	0.000	0.871
*SE*	0.173	0.259	0.213	0.148	0.147	0.216	0.213	0.225	0.176
CI	0–0.536	0–0.789	0–0.764	0–0.470	0–0.435	0–0.734	0–0.677	0–0.706	0.344–0.965
*p*	.121	.304	.166	.185	1.000	**.030**	.226	1.000	**.002**
Total trip duration									
*R*	0.000	0.044	0.000	0.157	0.000	0.391	0.034	0.000	0.679
*SE*	0.094	0.173	0.142	0.153	0.165	0.234	0.136	0.204	0.287
CI	0–0.295	0–0.548	0–0.709	0–0.471	0–0.521	0–0.756	0–0.432	0–0.659	0–0.937
*p*	1.000	1.000	1.000	.223	1.000	.086	.495	1.000	.160

Note

Significant values are bolded.

Abbreviations: CI, confidence interval; *SE*, standard error.

### 3.3 Intra‐ and interindividual spatial similarity of foraging flights

We found that interindividual home range (i.e., the 95% utilization densities) overlap ranged from 0.00 to 0.89 (all trips by both sexes analyzed). We recorded the highest interindividual overlap at Bydgoszcz (mean 0.26), and the lowest at Przykona (0.09). The proportion of cases of high (BA for >0.75) interindividual home range overlap was generally very low, ranging from 0.5% at Przykona to 5.6% at Bydgoszcz and 7.1% at Koronowo (Table 4). We found a relatively high intersex home range overlap of trips performed by BHGs (0.69 at Koronowo, 0.79 at Bydgoszcz, 0.84 at Przykona).

**TABLE 4:** Similarity of intra‐ and interindividual utilization densities (Bhattacharyya's affinity for 95% utilization densities [BA]) of GPS logger‐equipped black‐headed gulls from the studied colonies (BYD—Bydgoszcz; KOR—Koronowo; PRZ—Przykona) during the incubation period

Variable	Intraindividual BA			Interindividual BA			Intrafemale BA			Intramale BA		
	BYD	KOR	PRZ	BYD	KOR	PRZ	BYD	KOR	PRZ	BYD	KOR	PRZ
Min	0.13	0.06	0.04	0.00	0.00	0.00	0.32	0.06	0.04	0.13	0.83	0.18
Max	0.70	0.88	0.81	0.79	0.89	0.85	0.60	0.80	0.81	0.70	0.88	0.72
Mean	0.47	0.67	0.39	0.26	0.19	0.09	0.46	0.53	0.38	0.48	0.85	0.42
*SD*	0.24	0.29	0.30	0.23	0.24	0.15	0.19	0.33	0.34	0.30	0.02	0.27
%BA > 0.75	0.0	57.1	12.5	5.6	7.1	0.5	0.0	25.0	20.0	0.0	100.0	0.0
*N* trips	11	14	19	71	183	216	5	8	10	6	6	9
*N* individuals	5	7	8	8	13	10	2	4	5	3	3	3

Note

Only trips with at least five GPS positions recorded were considered. *N* trips—number of compared trips of the same individual; %BA > 0.75—proportion (%) of individuals with high (with BA > 0.75) overlap in 95% utilization densities between consecutive flights.

Abbreviation:* SD*, standard deviation.

The intraindividual (i.e., for particular flights of the same individual) home range overlap (BA) ranged from 0.04 to 0.88 with the highest mean value at Koronowo (0.67), and the lowest at Przykona (0.39; Table 4). The proportion of individuals highly overlapping in 95% UD (BA > 0.75) during the consecutive flights was relatively high at Koronowo (57%) and considerably lower in other colonies, that is, 12% in Przykona and no cases at Bydgoszcz (Table 4). We did not found significant differences between intraindividual home ranges overlap between females (0.45 ± 0.30, *n* = 11 individuals) and males (0.59 ± 0.29, *n* = 9 individuals; *t* = 1.03, *p* = .32).

Values of mean home ranges overlap, and the proportion of individuals highly overlapping in 95% UD in intraindividual comparisons was higher than in the case of interindividual comparison, which indicates a considerable level of foraging site fidelity (Table 4).

**FIGURE 4:**
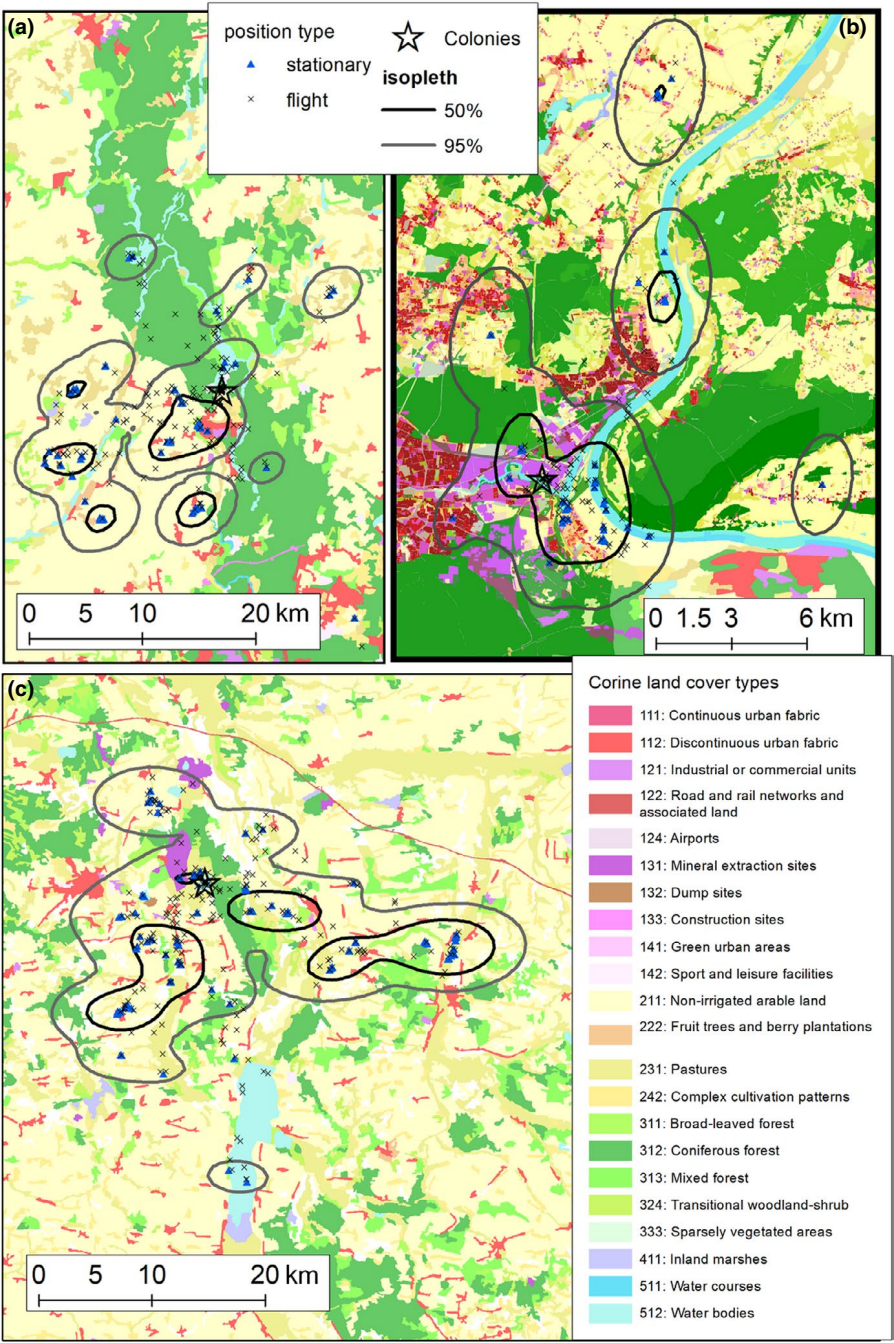
Utilization densities (UDs; 95% kernel home ranges and 50% core ranges) of black‐headed gulls during the incubation period. (a) UDs of birds breeding at Koronowo. (b) the core ranges of black‐headed gulls breeding in a highly urbanized area in Bydgoszcz. (c) UDs for birds breeding at Koronowo. Maps created based on data from CORINE land cover model CLC2018 (https://land.copernicus.eu/paneuropean/corine-land-cover/clc2018) (a and c) and Urban Atlas 2012 (http://land.copernicus.eu/local/urban-atlas/urban-atlas-2012/view) (b)

### 3.4 Intercolony differences in habitat use

The analysis of habitat use was limited to three habitat types (i.e., agricultural areas, artificial surfaces, and water bodies) that were associated with the majority (>96%) of recorded foraging positions. Habitat use differed significantly between the colonies (*χ* ^2^ = 122.1, *p* < .001). Birds from the urban colony at Bydgoszcz used agricultural areas significantly less frequently than birds from the rural colonies at Koronowo and Przykona, while BHGs from the rural colony in Przykona used artificial surfaces significantly less frequently than birds from Bydgoszcz and Koronowo (Table 5; Figure 4). BHGs from Bydgoszcz used water bodies significantly more frequently than birds from Bydgoszcz and Koronowo (Table 5; Figure 4).

**TABLE 5:** Habitat use and selection by GPS logger‐equipped black‐headed gulls during the incubation period at three different colonies measured using Manly's compositional analysis

*Bydgoszcz*	Avail.	Used	Wi	*SE*	IClower	ICupper
AGRIC	0.67	0.21	*0.31*	0.11	0.05	0.57
ARTIF	0.29	0.47	1.63	0.46	0.53	2.73
WATBOD	0.04	0.32	**8.34**	2.87	1.47	15.22
(*N* = 10 ind.)	Value	*df*	*p*	Intercolony differences		
Khi2L1 (A)	11.33	18	.880	AGRIC	^a,b^	
Khi2L2 (B)	24.79	20	.210	ARTIF	^a^	
B‐A	13.46	2	.001	WATBOD	^a,b^	

*Koronowo*	Avail.	Used	Wi	*SE*	IClower	ICupper
AGRIC	0.68	0.58	0.84	0.18	0.40	1.28
ARTIF	0.23	0.15	0.66	0.36	−0.20	1.53
WATBOD	0.08	0.27	3.27	1.45	−0.20	6.74
(*N* = 13 ind.)	Value	*df*	*p*	Intercolony differences		
Khi2L1 (A)	20.22	24	.684	AGRIC	^a^	
Khi2L2 (B)	24.27	26	.560	ARTIF	^b^	
B‐A	4.05	2	.132	WATBOD	^a^	

*Przykona*	Avail.	Used	Wi		*SE*	IClower	ICupper
AGRIC	0.81	0.87	1.08		0.11	0.80	1.35
ARTIF	0.18	0.01	*0.06*		0.06	−0.09	0.22
WATBOD	0.01	0.12	8.97		6.88	−7.49	25.43
(*N* = 11 ind.)	Value	*df*		*p*	Intercolony differences		
Khi2L1 (A)	7.45	20		.995	AGRIC	^b^	
Khi2L2 (B)	13.99	22		.902	ARTIF	^a,b^	
B‐A	6.54	2		.038	WATBOD	^b^	

Note

Avail.—proportion of three main habitat types (according to CORINE land cover model CLC2018, level 1: AGRIC—agricultural areas; ARTIF—artificial surfaces; WATBOD—water bodies) available in a 27‐km buffer around the studied colonies; Used—proportion of three main habitat types in stationary positions of GPS‐tracked individuals during the incubation period; Wi—habitat selection ratio; *SE*—standard error of Wi; IClower and ICupper—5%–95% confidence intervals for Wi. Values of Wi indicating significant habitat selection (with the lower CI limit above 1) are bolded. Values of Wi indicating significant habitat avoidance (with the upper CI limit below 1) are italicized; Khi2L1 (A)—results of the test of identical use of habitat by all animals; Khi2L2 (B)—results of the test of overall habitat selection; B‐A—results of the test of the hypothesis that animals are on average using resources in proportion to availability, irrespective of whether they are the same or not (i.e., Khi2L2–Khi2L1); Intercolony differences—differences in the proportion of particular habitats used.

^a,b^ Significant intercolony differences in habitat use (chi‐square test, *p* > .05).

We found that individuals from the colony at Bydgoszcz and Przykona were significantly selective; that is, they used habitats not proportionally to their availability in the vicinity (Khi2L2–Khi2L1, *p* = .001 and *p* = .04, respectively; Table 5). In contrast, birds from Koronowo used habitats proportionally to their availability in the area (*p* = .13). BHGs from the colony at Bydgoszcz showed a significant preference toward water bodies and avoided agricultural areas, while birds from Przykona avoided artificial surfaces (Table 5).

### 3.5 Sex differences in habitat use

**FIGURE 5:**
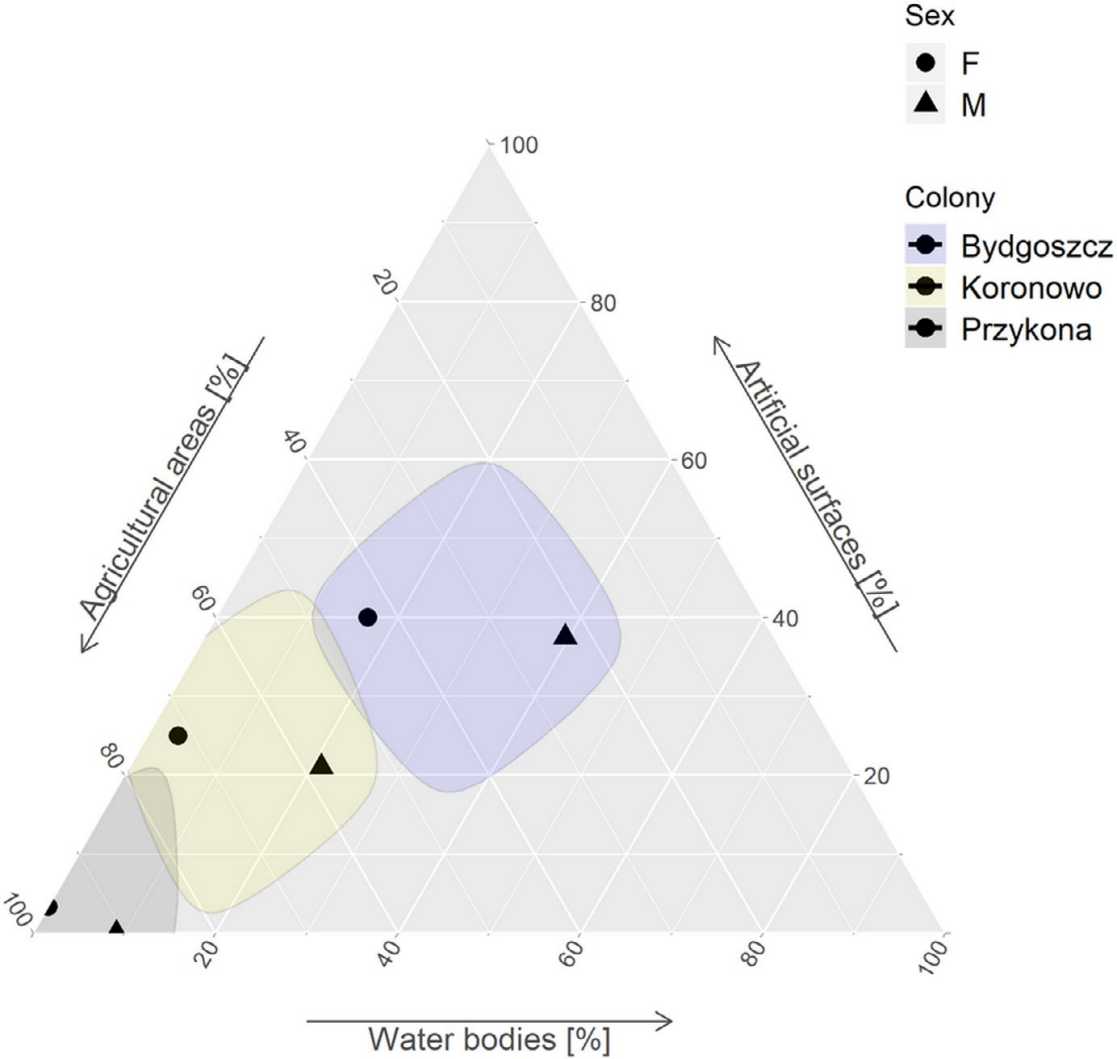
Ternary plot presenting relative proportions of three habitats types (CLC2018 level 1): agricultural areas, artificial surfaces, and water bodies in foraging positions of males and females of GPS logger‐equipped black‐headed gulls during the incubation period (all individuals of the same sex combined)

We found that males from the two rural colonies (Przykona and Koronowo) used water bodies significantly more frequently than females (Table 6; Figure 5). We found total habitat partitioning for both sexes at Przykona with females not using water bodies and males artificial surfaces (Table 6; Figure 5). In all study colonies, males used habitats unproportionally to their availability in the vicinity (Khi2L2–Khi2L1, all *p* < .03). In contrast, females from all studied colonies used habitats proportionally to their availability (all *p* > .37; Table 6). Males from Bydgoszcz had a significant preference toward water bodies and avoided agricultural areas, while males from Przykona avoided artificial surfaces (Table 6). Agricultural areas were significantly preferred by females from Przykona. The artificial surfaces were avoided by birds of both sexes from this colony, while water bodies only by females (Table 6).

**TABLE 6:** Habitat use and selection by GPS logger‐equipped females and males of black‐headed gulls during the incubation period at three different colonies measured using Manly's compositional analysis

Variable	Used	Wi	*SE*	IClower	ICupper	Used	Wi	*SE*	IClower	ICupper
*Bydgoszcz females (N = 3 ind.)*						*Bydgoszcz males (N = 7 ind.)*				
AGRIC	0.30	0.45	0.30	−0.27	1.17	0.16	*0.24*	0.10	0.002	0.49
ARTIF	0.53	1.83	0.82	−0.14	3.81	0.44	1.54	0.60	0.12	2.97
WATBOD	0.17	4.35	3.57	−4.21	12.90	0.39	**10.05**	3.77	1.03	19.08
Tests	Value	*df*	*p*			Value	*df*	*p*		
Khi2L1 (A)	2.32	4	.677			8.44	12	.750		
Khi2L2 (B)	4.28	6	.639			20.51	14	.115		
B‐A	1.96	2	.376			12.08	2	**.002**		
*Koronowo females (N = 7 ind.)*						*Koronowo males (N = 6 ind.)*				
AGRIC	0.72	1.06	0.22	0.54	1.57	0.40	0.59	0.29	−0.11	1.29
ARTIF	0.21	0.88	0.59	−0.54	2.30	0.10	0.41	0.41	−0.57	1.38
WATBOD^a^	0.07	0.87	0.69	−0.78	2.51	0.50	6.06	2.71	−0.43	12.56
Tests	Value	*df*	*p*			Value	*df*	*p*		
Khi2L1 (A)	7.11	12	.851			9.87	10	.452		
Khi2L2 (B)	7.16	14	.929			17.11	12	.145		
B‐A	0.05	2	.975			7.24	2	**.027**		
*Przykona females (N = 6 ind.)*						*Przykona males (N = 5 ind.)*				
AGRIC	0.98	**1.21**	0.03	1.15	1.28	0.73	0.91	0.24	0.33	1.49
ARTIF	0.02	*0.12*	0.12	−0.17	0.40	0.00	*0.00*	0.00	0.00	0.00
WATBOD^a^	0.00	*0.00*	0.00	0.00	0.00	0.27	19.73	14.38	−14.70	54.17
Tests	Value	*df*	*p*			Value	*df*	*p*		
Khi2L1 (A)	0.48	10	1.000			4.53	8	.807		
Khi2L2 (B)	2.21	12	.999			11.79	10	.300		
B‐A	1.73	2	.421			7.26	2	**.027**		

Note

See codes and explanations in Table 5.

^a^ Significant intersex differences in habitat use (Fisher's exact test, *p* < .04).

### 3.6 Diversity of habitat use

We found significant differences in the diversity of habitat use (DHU) by birds from different colonies (Kruskal–Wallis test, *p* = .027). Despite the same median DHU for all colonies (0.98), foraging flights of birds from Bydgoszcz (range: 0.33–0.99, IQR = 0.51, *N* = 26 flights by 10 birds) were characterized by a significantly lower DHU values compared to Przykona (range: 0.41–0.99, IQR = 0.0, *N* = 24 flights by 11 birds; Wilcoxon test, *p* = .023) and marginally nonsignificantly lower values compared to Koronowo (range: 0.40–0.99, IQR = 0.0, *N* = 24 flights by 13 birds; *p* = .053). DHU values in both rural colonies were similar (*p* = .65). These results indicate relatively high consistency in habitat use during particular flights of individuals from all colonies. No intersex differences in DHU were recorded when data from all the colonies were combined (Wilcoxon test, *p* = .86) or analyzed separately (all *p* > .13).

BHGs from our study populations varied substantially in habitat specialization (along the generalist–specialist spectrum). We found that 62% individuals from Bydgoszcz (*N* = 8), 86% from Koronowo (*N* = 7), and 100% from Przykona (*N* = 9) foraged predominantly in a single habitat type (i.e., >95% foraging positions from all flights were recorded in one type of habitat). We found that birds from rural colonies mostly specialized in exploiting agricultural areas (83% of individuals and 71.9% of all habitat specialists in Koronowo; 100% of individuals in Przykona). In the urban colony at Bydgoszcz, 37.5% of all individuals (60% of all habitat specialists) specialized in exploiting artificial surfaces; among the remaining individuals from this colony, one specialized in water bodies and another one in agricultural areas.

### 3.7 Relationships between habitat use and foraging flight characteristics

**FIGURE 6:**
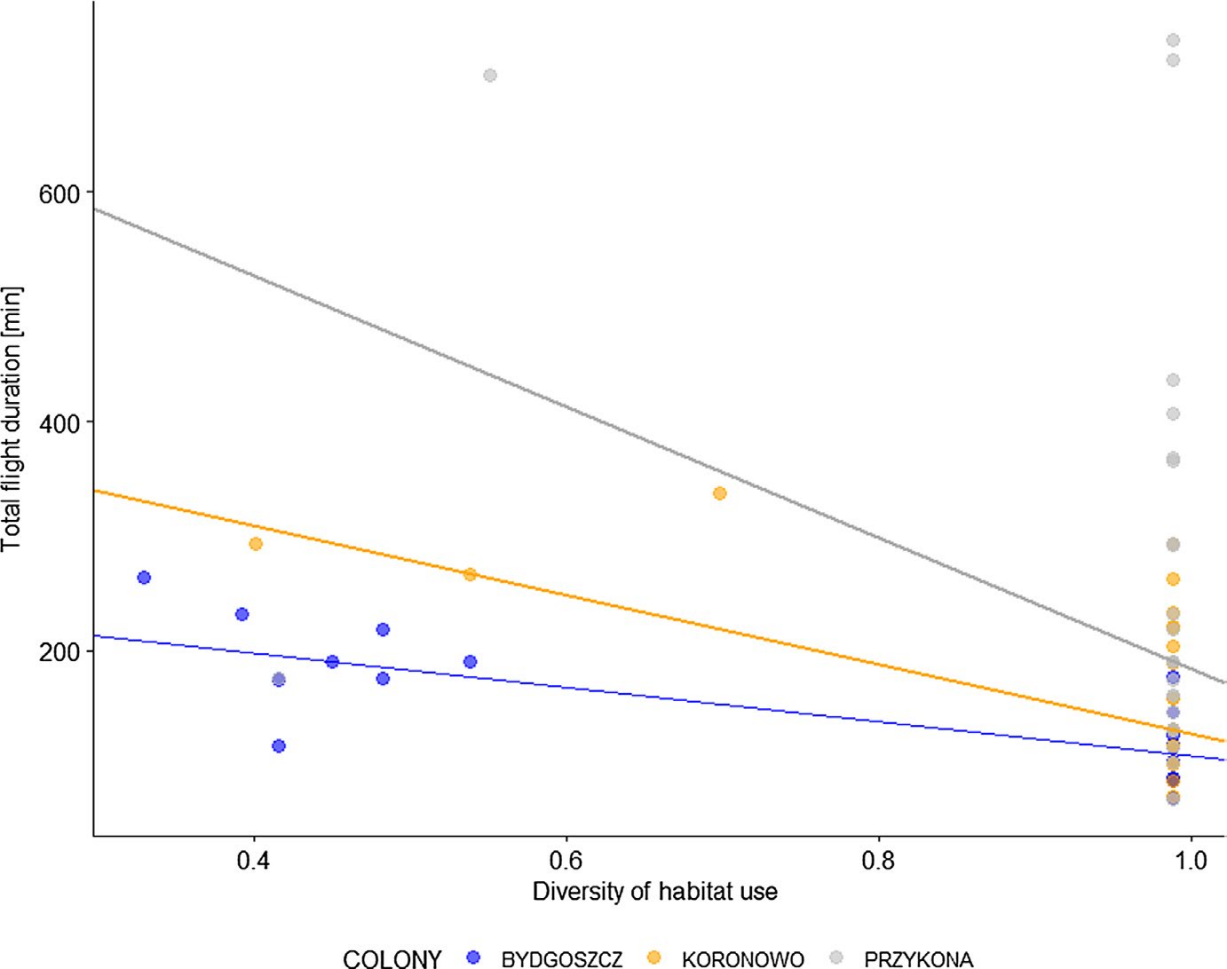
Relationship between diversity habitat use and total flight duration. Lines indicate significant relationships (determined by Kendall‐Theil Sen‐Siegel nonparametric linear regression)

We found a positive significant relationships between diversity of habitat use and total flight duration both when data from all the colonies were combined (Kendall–Theil Sen–Siegel test, MAD = 125.5, *p* < .001) and analyzed separately (Bydgoszcz, MAD = 60.5, *p* < .001; Koronowo, MAD = 97.2, *p* < .001; Przykona, MAD = 243.3.2, *p* < .001). The total flight duration decreased with increasing diversity of habitat use, meaning that habitat specialists spent less time foraging than habitat generalists (Figure 6). We found no significant relationship between diversity habitat use and total distance covered (MAD = 29.07, *p* = .113).

**FIGURE 7:**
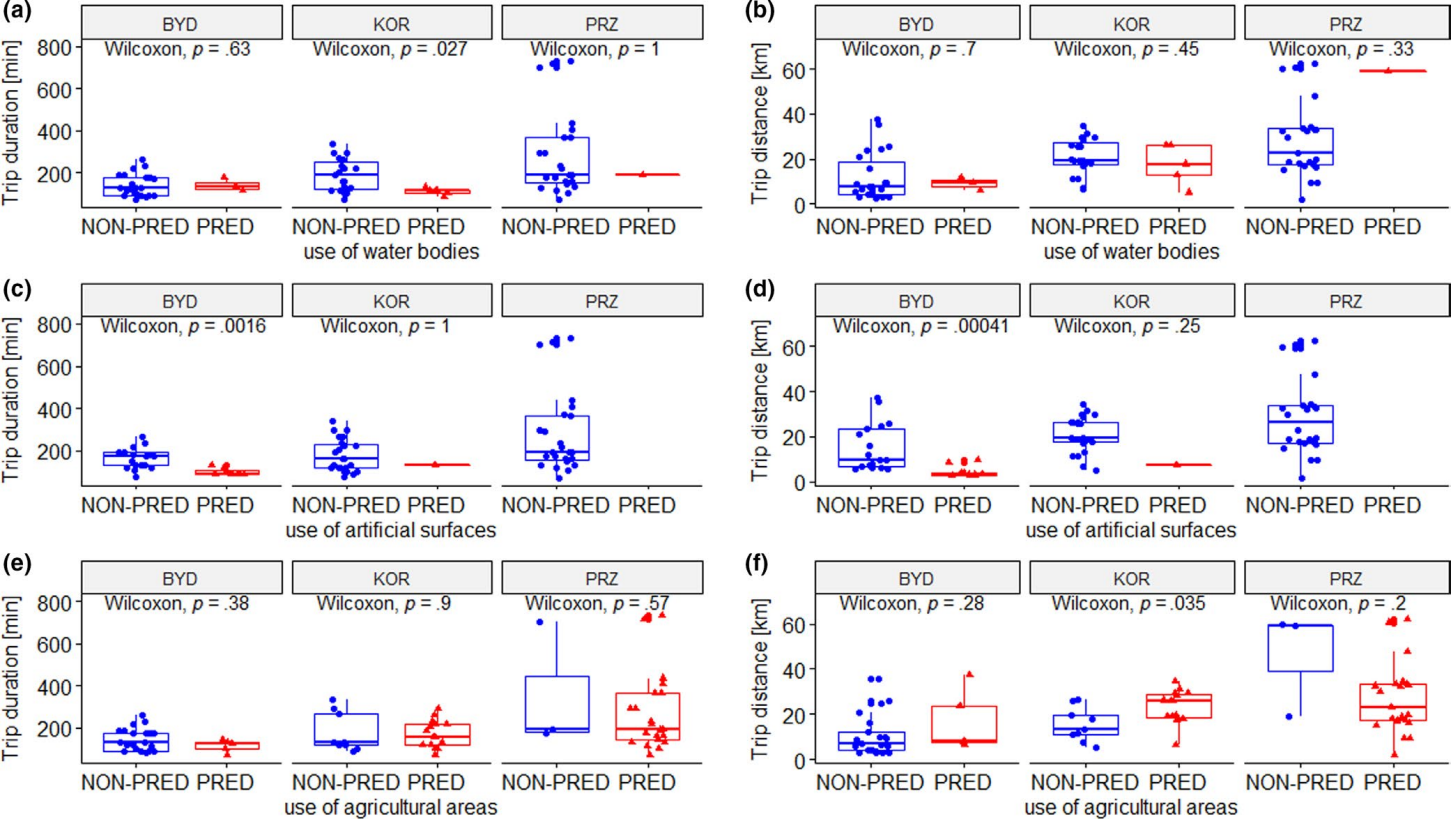
Relationship between use of particular habitats (PRED—predominant relative ratio (>95%) of foraging position in one type of habitat; NON‐PRED— nonpredominant relative ratio (≤95%) of foraging position in one type of habitat) and total flight duration (a, c, e) and total distance covered (b, d, e) of birds breeding in the colonies at Bydgoszcz (BYD), Koronowo (KOR), and Przykona (PRZ)

Specifically, we found that individuals from Koronowo that specialized in exploiting water bodies performed significantly shorter flights than specialists foraging in artificial areas and habitat generalists from this colony. Also, individuals from Koronowo that specialized in exploiting agricultural areas covered greater distances compared to individuals not specialized in the use of this habitat (Figure 7). BHGs from the urban colony at Bydgoszcz that specialized in exploiting artificial surfaces spent less time and covered smaller distances compared to individuals not specialized in this habitat type (Figure 7).

## 4 DISCUSSION

Our study is the first to show maximal foraging distances of BHG during the incubation period based on precise data from GPS loggers. A study on one radio‐tagged BHG individual from north Bavaria, Germany, tracked during the incubation period revealed that it performed only short flights <5 km lasting up to one hour (Gorke & Brandl, 1986). The maximal distance of foraging trips found in the colonies at Bydgoszcz and Koronowo (17.3 and 14.3 km, respectively) were more similar to distances of up to 18.5 km reported for the chick‐rearing radio‐tagged BHGs breeding in north Bavaria, Germany (Brandl & Gorke, 2007; Gorke & Brandl, 1986). To our knowledge, the maximal distance recorded at Przykona (26.7 km) was the highest ever reported for this species during any reproductive stage.

### 4.1 Intercolony differences

As we expected, characteristics of foraging flights of GPS logger‐equipped BHGs performed during the incubation period differed between the colonies. Birds from the urban colony in Bydgoszcz performed the shortest trips (as measured with maximal range and total flight distance) compared to individuals from rural colonies, especially at Przykona. Birds from the Bydgoszcz colony have many attractive feeding grounds including riverine habitats, agricultural areas, sewage treatment plants and oxbow lakes in cost‐effective distance from the colony (Figure 4). Birds from the rural colony at Przykona performed the longest flights in terms of time and distance and it was well reflected by the highest slope value for the relationship between the total distance covered and total flight duration (Figure 3). These intercolony differences in flight characteristics may be explained in terms of various habitat selections. The differences in foraging flight ecology were highly apparent between Bydgoszcz and Przykona; birds from these colonies were highly selective; that is, they used habitats not proportionally to their availability.

Aquatic habitats serve as important foraging areas of BHGs providing nutritionally beneficial food, such as fish—the prey high in calories and protein and containing high levels of calcium and sulfonated amino acids (Pierotti & Annett, 1987). Indeed, birds from all our study colonies most frequently foraged at water bodies and individuals from Bydgoszcz significantly selected only this type of habitat, which may be explained by the specific location of the colony. BHGs from Bydgoszcz bred on the islet in a small artificial bay of the eutrophic Brda River in a close distance to the Vistula River and these habitats offer easy access to abundant aquatic prey. Although some GPS positions that we classified as foraging at water bodies may indeed reflect resting or bathing, our direct observations indicate that active foraging was common in this type of habitat. Birds from the two rural colonies used water bodies less frequently than birds from Bydgoszcz. The colony at Koronowo was located on a large mesotrophic reservoir, which is regularly used for recreation (water sports), and seasonal holiday houses are located near the island. Thus, other habitats may be more attractive for a majority of birds from this colony, but we still found that 20% of individuals specialized in exploiting water bodies. BHGs from Przykona, despite breeding at a relatively large water reservoir, used this type of habitat with the lowest frequency among our study colonies. Sympatric breeding with two other gull species (Mediterranean and Caspian gulls) may increase competition over local food resources in the vicinity of the Przykona colony. Thus, the majority of birds probably foraged in other habitats. Individuals from this colony were less frequently recorded foraging at their breeding reservoir compared to other water bodies. Some individuals flew to forage in a distant (17 km from the colony), but food‐abundant (eutrophic), large (4,230 ha) Jeziorsko reservoir (Figure 2). One may expect that birds should breed in close distance to attractive food supply to optimize the costs of foraging. Flights of birds from Przykona toward distant foraging grounds combined with the results of a previous study from the North Sea coast (intercolony differences in BHG diet not concordant with the location of potential foraging areas; Schwemmer & Garthe, 2008) indicate that high quality of foraging habitat can possibly outweigh the costs of long foraging flights in this species. Alternatively, it may be a consequence of locally limited availability of attractive breeding sites.

The most frequent use of agriculture habitats by birds from rural colonies at Koronowo and Przykona may result from the low availability of alternative attractive feeding areas in the cost‐effective distance from the colony. BHGs may feed in such habitats mainly on invertebrates—earthworms and beetles (e.g., Cuendet, 1983; Vernon, 1972b)—and foraging in agriculture areas has been also reported for other areas, for example, coastal lowland landscape of Germany (Schwemmer, Garthe, & Mundry, 2008).

Birds from the urban colony at Bydgoszcz most frequently used artificial surfaces for foraging. It has been previously reported that gulls from urban colonies at Bydgoszcz regularly exploited human‐derived food resources, as assessed from the occurrence of anthropogenic food remains in regurgitated pellets and frequent observations of gulls feeding on municipal landfill site at Bydgoszcz (Indykiewicz, Podlaszczuk, Janiszewska, & Minias, 2018). Urban BHGs breeding at Bydgoszcz were also reported to be bolder and more aggressive toward humans compared to birds from rural colonies (Indykiewicz et al., 2018), which makes them well adapted to an exploitation of anthropogenic food sources in the urban environment. In contrast, birds from the rural colony at Przykona avoided foraging close to human settlements and the inspection of pellets regurgitated in rural colonies provided no evidence for the use of anthropogenic food (Indykiewicz et al., 2018). A higher use of artificial surface by birds from Koronowo compared to the other rural colony at Przykona may be explained by the location of the former colony in the close proximity of the town where BHGs may have foraged on anthropogenic‐origin food.

### 4.2 Sex differences

BHG males and females covered similar distances during their foraging flights. However, females in all colonies spent significantly more time on foraging flights compared to males. The flights of females, in contrast to males, were characterized by a lack of significant repeatability of maximal flight range or total distance covered despite the fact that they generally explored similar areas (high overlap in home ranges). These differences in flight duration and repeatability of flight characteristics may have resulted from sex differences in habitat use and selection. Between‐sex differences in habitat use have been also found in other gulls, but mainly in larger species (e.g., Camphuysen et al., 2015; Kazama et al., 2018; Navarro et al., 2010; Pons, 1994).

Generally, females from all study colonies were less selective in habitat use than males—they used particular habitats proportionally to their availability in the area. Foraging in different habitats on different prey may require various competitive and cognitive abilities, which may affect flight duration. Larger body size of males (14% higher body mass and 5%–11% larger morphometric measurements; Indykiewicz et al., 2019) may predispose them to the energetically more demanding and more competitive foraging. We found significant repeatability in the maximal range and total distance covered during foraging flights only in males, which may indicate that smaller females were less site‐faithful and displaced from attractive foraging habitats more frequently than males.

Males from both rural colonies used water bodies more frequently than females. In the urban colony at Bydgoszcz water bodies were significantly selected by males but not females. These preferences may reflect varying competitive abilities of males and females in food‐abundant habitats associated with strong inter‐ and intraspecific competition. Also, female lesser black‐backed gulls *Larus fuscus* that were GPS‐tracked during foraging in the Wadden Sea spent more time foraging on land, while larger males mostly foraged at the open North Sea (Camphuysen et al., 2015). In black‐tailed gulls *Larus crassirostris* breeding in Japan, larger males frequently foraged in fishing ports and fish processing plants, whereas smaller females rarely foraged in these habitats (Kazama et al., 2018).

Foraging in agricultural areas (positively selected by females at Przykona and avoided by males at Bydgoszcz) is usually not associated with prey capturing during mass feeding frenzies under strong inter‐ or intraspecific competition that would require particular competitive strength. Most natural food items on agricultural areas may be taken rather circumstantially, under the influence of particular weather conditions (insects and earthworms) or agricultural activities (small mammals, cereals, insects, and worms; Camphuysen et al., 2015). Thus, this habitat may be used more frequently by less competitive females.

Surprisingly, in the rural colony at Przykona exclusively females used artificial surfaces, that is, urbanized habitats which may potentially require higher competitive abilities. In contrast, the study on herring gulls *Larus argentatus* revealed sex differences in diet composition: Females ate more earthworms and less garbage than males (Pons, 1994) and these differences were linked to the high level of aggression at the landfill, which made less competitive females shift to alternative feeding grounds. On the other hand, we cannot be sure whether females from the second rural colony, Koronowo, foraged on the food sources requiring high competitive abilities. GPS positions in the urban zone at Koronowo town were recorded mainly in the center of town close to the river where birds may have foraged on aquatic prey or food provided opportunistically by pedestrians.

### 4.3 Individual specialization

As we expected, an individual component was clearly visible in foraging flight strategies at a colony scale. It was indicated by low interindividual and high intraindividual overlap of home ranges. Within‐individual component explained a considerable part of the total variance in utilization density overlaps. Maximal flight ranges were or tended to be repeatable (within individuals) in all colonies. Radio‐tagged BHGs from Bavaria also showed considerable individual components in flights (Gorke & Brandl, 1986). Our study birds varied substantially in habitat specialization, but the vast majority (86%–100%) of individuals from both rural colonies foraged predominantly in one specific type of habitat.

Individual spatial specialization may have a selective advantage in environments where resources are predictable. Gulls from Koronowo were characterized by the relatively highest proportion (57%) of individuals with high overlap of home ranges (i.e., with BA > 0.75) between the consecutive flights. Relationships between the use of predominant habitat type and foraging flight characteristics suggest that some birds were faithful to a specific habitat type despite potentially higher time investments (longer duration of flights toward artificial surfaces) or energy expenditures (further distance to agricultural areas). It is possible that these habitats offered predictable and attractive food, despite higher costs of arrival at foraging sites. In such conditions, returning to the same foraging ground in consecutive flights may be advantageous. The study on herring gulls breeding in the Belgian coastal habitats revealed that individuals with higher levels of habitat specialization covered on average shorter daily distances, which was associated with certain fitness benefits, as chicks of habitat specialists grew faster (van den Bosch, Baert, Müller, Lens, & Stienen, 2019).

In the urban colony at Bydgoszcz, we found no cases of high home range overlap, but we recorded the lowest number of individuals specialized in the specific habitat (62%), which may be explained by more opportunistic feeding in the city. However, similarly to other colonies, foraging flights of habitat specialists lasted shorter compared to flights of birds that used diverse habitats. Although we do not know the exact foraging success rate associated with each habitat type, it seems safe to assume that birds are likely to return to their colony after successful foraging, which may indicate higher foraging success of habitat specialists.

## 5 CONCLUSIONS

This study highlights that black‐headed gulls, which are considered to be generalists, can be remarkably consistent in their foraging behavior. We found considerable interindividual differences in foraging flight characteristics and habitat use. However, many individuals were highly repeatable in home range and habitat use. These interindividual differences and intraindividual similarities probably reflect phenotypic plasticity both in foraging strategies and boldness (Indykiewicz et al., 2018) and allow this species to exploit a wide spectrum of habitats, including highly urbanized areas that require higher competitive abilities. Intersex differences in the foraging flight duration and habitat use may be attributed to selective foraging on food sources requiring various competitive abilities. Further studies, ideally supported by diet composition data (e.g., stable isotope ratios, pellet composition), are needed to better comprehend the foraging ecology of black‐headed gulls.

## CONFLICT OF INTERESTS

The authors declare no conflicts of interests.

## AUTHOR CONTRIBUTION

**Dariusz Jakubas:** Conceptualization (lead); Data curation (lead); Formal analysis (lead); Funding acquisition (lead); Methodology (lead); Resources (lead); Visualization (lead); Writing‐original draft (lead); Writing‐review & editing (equal). **Piotr Indykiewicz:** Conceptualization (lead); Investigation (lead); Resources (supporting); Supervision (lead); Writing‐review & editing (equal). **Jarosław Kowalski:** Investigation (lead); Writing‐review & editing (supporting). **Tomasz Iciek:** Investigation (lead); Writing‐review & editing (supporting). **Piotr Minias:** Conceptualization (lead); Investigation (lead); Resources (supporting); Supervision (lead); Writing‐review & editing (equal). 

## Data Availability

Positions of GPS‐tracked black‐headed gulls, characteristics of individuals and their flights, and types of land cover: Dryad https://doi.org/10.5061/dryad.q83bk3jfg.

## References

[ece36291-bib-0001] Andersson, M. , Götmark, F. , & Wiklund, C. G. (1981). Food information in the Black‐headed Gull, *Larus ridibundus* . Behavioral Ecology and Sociobiology, 9, 199–202.

[ece36291-bib-0002] Barta, Z. (2001). Breeding colonies as information centers: A reappraisal of information‐based hypotheses using the producer–scrounger game. Behavioral Ecology, 12, 121–127.

[ece36291-bib-0003] Blount, J. D. , Houston, D. C. , Surai, P. F. , & Møller, A. P. (2004). Egg‐laying capacity is limited by carotenoid pigment availability in wild gulls *Larus fuscus* . Proceedings of the Royal Society B: Biological Sciences, 271, 79–81. 10.1098/rsbl.2003.0104 PMC1809998 15101425

[ece36291-bib-0004] Bolnick, D. I. (2001). Intraspecific competition favours niche width expansion in *Drosophila melanogaster* . Nature, 410, 463–466. 11260712 10.1038/35068555

[ece36291-bib-0005] Bolnick, D. I. , & Kirkpatrick, M. (2012). The relationship between intraspecific assortative mating and reproductive isolation between divergent populations. Current Zoology, 58, 484–492.

[ece36291-bib-0006] Bolton, M. , Conolly, G. , Carroll, M. , Wakefield, E. D. , & Caldow, R. (2019). A review of the occurrence of inter‐colony segregation of seabird foraging areas and the implications for marine environmental impact assessment. Ibis, 161, 241–259.

[ece36291-bib-0007] Brandl, R. , & Gorke, M. (2007). How to live in colonies: Foraging range and patterns of density around a colony of black‐headed gulls *Larus ridibundus* in relation to the Gulls’ Energy Budget. Ornis Scandinavica, 19, 305.

[ece36291-bib-0008] Brandl, R. , & Nelsen, I. (1988). Feeding frequency of black‐headed gull chicks. Bird Study, 35, 137–141.

[ece36291-bib-0009] Calenge, C. (2006). The package “adehabitat” for the R software: A tool for the analysis of space and habitat use by animals. Ecological Modelling, 197, 516–519.

[ece36291-bib-0010] Calenge, C. (2011). Exploratory analysis of the habitat selection by the wildlife in R: The “adehabitatHS” Package (vignette), 1–60.

[ece36291-bib-0011] Camphuysen, K. C. J. , Shamoun‐Baranes, J. , Van Loon, E. E. , & Bouten, W. (2015). Sexually distinct foraging strategies in an omnivorous seabird. Marine Biology, 162, 1417–1428.

[ece36291-bib-0012] Cleasby, I. , Wakefield, E. , Bodey, T. , Davies, R. , Patrick, S. , Newton, J. , … Hamer, K. (2015). Sexual segregation in a wide‐ranging marine predator is a consequence of habitat selection. Marine Ecology Progress Series, 518, 1–12.

[ece36291-bib-0013] Cuendet, G. (1983). Predation on earthworms by the Black‐headed Gull (*Larus ridibundus* L.) In Earthworm ecology (pp. 415–424). Dordrecht, The Netherlands: Springer.

[ece36291-bib-0014] Dall, S. R. X. , Bell, A. M. , Bolnick, D. I. , & Ratnieks, F. L. W. (2012). An evolutionary ecology of individual differences (A Sih, Ed.). Ecology Letters, 15, 1189–1198. 22897772 10.1111/j.1461-0248.2012.01846.x PMC3962499

[ece36291-bib-0015] Elliott, K. H. , Davoren, G. K. G. K. , & Gaston, A. J. (2007). The influence of buoyancy, and drag on the dive behaviour of an Arctic seabird, the Thick‐billed Murre. Canadian Journal of Zoology, 85, 352–361.

[ece36291-bib-0016] Enners, L. , Schwemmer, P. , Corman, A. M. , Voigt, C. C. , & Garthe, S. (2018). Intercolony variations in movement patterns and foraging behaviors among herring gulls (*Larus argentatus*) breeding in the eastern Wadden Sea. Ecology and Evolution, 8, 7529–7542. 30151168 10.1002/ece3.4167 PMC6106178

[ece36291-bib-0017] Estévanez, C. A. B. , & Aparicio, S. P. (2019). Competitive inter‐ and intraspecific dominance relations in three gull species. Revista Catalana D'ornitologia, 35, 21–29.

[ece36291-bib-0018] Garriga, J. , Palmer, J. R. B. , Oltra, A. , & Bartumeus, F. (2016). Expectation‐maximization binary clustering for behavioural annotation. PLoS ONE, 11, 1–26. 10.1371/journal.pone.0151984 PMC4803255 27002631

[ece36291-bib-0019] González‐Solís, J. , Phillips, R. A. , Daunt, F. , Lewis, S. , & Wilson, R. P. (2017). THEME: Individual variability in seabird foraging and migration. Marine Ecology Progress Series, 578, 115–261.

[ece36291-bib-0020] Gorke, M. , & Brandl, R. (1986). How to live in colonies: Spatial foraging strategies of the black‐headed gull. Oecologia, 70, 288–290. 28311671 10.1007/BF00379253

[ece36291-bib-0021] Gotmark, F. (1984). Food and foraging in five European *Larus gulls* in the breeding season: A comparative review. Ornis Fennica, 61, 9–18.

[ece36291-bib-0022] Greig, S. A. , Coulson, J. C. , & Monaghan, P. (1985). Feeding strategies of male and female adult herring gulls (*Larus argentatus*). Behaviour, 94, 41–59.

[ece36291-bib-0023] Griffiths, R. , Double, M. C. , Orr, K. , & Dawson, R. J. G. (1998). A DNA test to sex most birds. Molecular Ecology, 7, 1071–1075. 9711866 10.1046/j.1365-294x.1998.00389.x

[ece36291-bib-0024] Halekoh, U. , & Højsgaard, S. (2014). A Kenward‐Roger Approximation and Parametric Bootstrap Methods for Tests in Linear Mixed Models: The R package pbkrtest. Journal of Statistical Software, 59, 1–30.26917999

[ece36291-bib-0025] Hedd, A. , Montevecchi, W. A. , Phillips, R. A. , Fifield, D. A. , & Garthe, S. (2014). Seasonal sexual segregation by monomorphic sooty shearwaters *Puffinus griseus* reflects different reproductive roles during the pre‐laying period. PLoS ONE, 9, e85572. 24416429 10.1371/journal.pone.0085572 PMC3887055

[ece36291-bib-0026] Indykiewicz, P. , Minias, P. , Kowalski, J. , & Podlaszczuk, P. (2019). Shortcomings of discriminant functions: A Case study of sex identification in the black‐headed gull. Ardeola, 66, 361.

[ece36291-bib-0027] Indykiewicz, P. , Podlaszczuk, P. , Janiszewska, A. , & Minias, P. (2018). Extensive gene flow along the urban–rural gradient in a migratory colonial bird. Journal of Avian Biology, 49, 1–10.

[ece36291-bib-0028] Ismar, S. M. H. , Raubenheimer, D. , Bury, S. J. , Millar, C. D. , & Hauber, M. E. (2017). Sex‐specific foraging during parental care in a size‐monomorphic seabird, the Australasian Gannet (*Morus serrator*). Wilson Journal of Ornithology, 129, 139–147.

[ece36291-bib-0029] Juvaste, R. , Arriero, E. , Gagliardo, A. , Holland, R. , Huttunen, M. J. , Mueller, I. , … Wistbacka, R. (2017). Satellite tracking of red‐listed nominate lesser black‐backed gulls (*Larus* f. *fuscus*): Habitat specialisation in foraging movements raises novel conservation needs. Global Ecology and Conservation, 10, 220–230.

[ece36291-bib-0030] Kazama, M. T. , Watanuki, Y. , Kazama, K. , Tsukamoto, S. , Gonzalez, J. E. , & Nishizawa, B. (2018). Male and female Black‐tailed Gulls *Larus crassirostris* feed on the same prey species but use different feeding habitats. Journal of Ornithology, 159, 923–934.

[ece36291-bib-0031] Kubetzki, U. , & Garthe, S. (2003). Distribution, diet and habitat selection by four sympatrically breeding gull species in the south‐eastern North Sea. Marine Biology, 143, 199–207.

[ece36291-bib-0032] Mangiafico, S. (2016). Summary and analysis of extension program evaluation in R, version 1.15.0. https://rcompanion.org/handbook

[ece36291-bib-0033] Manly, B. F. J. , McDonald, L. L. , Thomas,D. L. , McDonald, T. L. , & Erickson, W. P. (2007). Resource selection by animals: Statistical design and analysis for field studies. Germany: Springer Science & Business Media.

[ece36291-bib-0034] Masello, J. F. , Mundry, R. , Poisbleau, M. , Demongin, L. , Voigt, C. C. , Wikelski, M. , & Quillfeldt, P. (2010). Diving seabirds share foraging space and time within and among species. Ecosphere, 1, 1‐28.

[ece36291-bib-0035] Maynard, L. D. , & Ronconi, R. A. (2018). Foraging behaviour of great black‐backed gulls *Larus marinus* near an urban centre in Atlantic Canada: Evidence of individual specialization from GPS tracking. Marine Ornithology, 46, 27–32.

[ece36291-bib-0036] Moreira, F. (2007). Diet of Black‐Headed Gulls *Larus ridibundus* on emerged intertidal areas in the Tagus Estuary (Portugal): predation or grazing? Journal of Avian Biology, 26, 277.

[ece36291-bib-0037] Navarro, J. , Grémillet, D. , Ramirez, F. J. , Afán, I. , Bouten, W. , & Forero, M. G. (2017). Shifting individual habitat specialization of a successful predator living in anthropogenic landscapes. Marine Ecology Progress Series, 578, 243–251.

[ece36291-bib-0038] Navarro, J. , Oro, D. , Bertolero, A. , Genovart, M. , Delgado, A. , & Forero, M. G. (2010). Age and sexual differences in the exploitation of two anthropogenic food resources for an opportunistic seabird. Marine Biology, 157, 2453–2459.

[ece36291-bib-0039] Noordhuis, R. , & Spaans, A. L. (1992). Interspecific competition for food between Herring *Larus argentatus* and Lesser Black‐backed gulls *L. fuscus* in the Dutch Wadden Sea Area. Ardea, 80, 115–132.

[ece36291-bib-0040] Palomares, L. E. , Arroyo, B. E. , Marchamalo, J. , Sainz, J. J. , & Voslamber, E. (1997). Sex‐ and age‐related biometric variation of Black‐headed Gulls *Larus ridibundus* in Western European populations. Bird Study, 44, 310–317.

[ece36291-bib-0041] Phillips, R. A. , Lewis, S. , González‐Solís, J. , & Daunt, F. (2017). Causes and consequences of individual variability and specialization in foraging and migration strategies of seabirds. Marine Ecology Progress Series, 578, 117–150.

[ece36291-bib-0042] Phillips, R. A. , McGill, R. A. R. , Dawson, D. A. , & Bearhop, S. (2011). Sexual segregation in distribution, diet and trophic level of seabirds: Insights from stable isotope analysis. Marine Biology, 158, 2199–2208.

[ece36291-bib-0043] Phillips, R. A. , Silk, J. R. D. , Phalan, B. , Catry, P. , & Croxall, J. P. (2004). Seasonal sexual segregation in two *Thalassarche albatross* species: Competitive exclusion, reproductive role specialization or foraging niche divergence? Proceedings of the Royal Society B‐Biological Sciences, 271, 1283–1291. 10.1098/rspb.2004.2718 PMC1691717 15306353

[ece36291-bib-0044] Pierotti, R. , Annett, C. (1987). Reproductive consequences of dietary specialization and switching in an ecological generalist In Kamil A. C. et al (Ed.), Foraging behavior (pp. 417–442). New York, NY: Plenum Press.

[ece36291-bib-0045] Pons, J. M. (1994). Feeding strategies of male and female herring gulls during the breeding season under various feeding conditions. Ethology Ecology & Evolution, 6, 1–12.

[ece36291-bib-0046] R Core Team (2018). R: A language and environment for statistical computing. Vienna, Austria: R Foundation for Statistical Computing.

[ece36291-bib-0047] Ramesh, T. , Kalle, R. , & Downs, C. T. (2016). Spatiotemporal variation in resource selection of servals: Insights from a landscape under heavy land‐use transformation. Journal of Mammalogy, 97, 554–567.

[ece36291-bib-0048] Ramos, R. , Ramírez, F. , Sanpera, C. , Jover, L. , & Ruiz, X. (2009). Diet of Yellow‐legged Gull (*Larus michahellis*) chicks along the Spanish Western Mediterranean coast: The relevance of refuse dumps. Journal of Ornithology, 150, 265–272.

[ece36291-bib-0049] Richner, H. , & Heeb, P. (1995). Is the information center hypothesis a flop? Advances in the Study of Behaviour, 24, 1–46.

[ece36291-bib-0050] Schwemmer, P. , & Garthe, S. (2008). Regular habitat switch as an important feeding strategy of an opportunistic seabird species at the interface between land and sea. Estuarine, Coastal and Shelf Science, 77, 12–22.

[ece36291-bib-0051] Schwemmer, P. , Garthe, S. , & Mundry, R. (2008). Area utilization of gulls in a coastal farmland landscape: Habitat mosaic supports niche segregation of opportunistic species. Landscape Ecology, 23, 355–367.

[ece36291-bib-0052] Stoffel, M. A. , Nakagawa, S. , & Schielzeth, H. (2017). rptR: Repeatability estimation and variance decomposition by generalized linear mixed‐effects models. Methods in Ecology and Evolution, 8, 1639–1644.

[ece36291-bib-0053] Svanbäck, R. , & Bolnick, D. I. (2007). Intraspecific competition drives increased resource use diversity within a natural population. Proceedings of the Royal Society B: Biological Sciences, 274, 839–844. 10.1098/rspb.2006.0198 PMC2093969 17251094

[ece36291-bib-0054] van den Bosch, M. , Baert, J. M. , Müller, W. , Lens, L. , & Stienen, E. W. M. (2019). Specialization reduces foraging effort and improves breeding performance in a generalist bird. Behavioral Ecology, 30, 792–800.

[ece36291-bib-0055] Van Donk, S. , Shamoun‐Baranes, J. , Bouten, W. , Van Der Meer, J. , & Camphuysen, K. C. J. (2019). Individual differences in foraging site fidelity are not related to time‐activity budgets in Herring Gulls. Ibis, 162, 429–445.

[ece36291-bib-0056] Vandenabeele, S. P. , Shepard, E. L. , Grogan, A. , & Wilson, R. P. (2012). When three per cent may not be three per cent; device‐equipped seabirds experience variable flight constraints. Marine Biology, 159, 1–14.

[ece36291-bib-0057] Vernon, J. D. R. (1972a). Feeding habitats and food of the Black‐headed and common gulls. Bird Study, 19, 173–186.

[ece36291-bib-0058] Vernon, J. D. R. (1972b). Feeding habitats and food of the black‐headed and common gulls. Part 1 – Feeding Habits. Bird Study, 17, 287–296.

[ece36291-bib-0059] Wanless, S. , & Harris, M. P. (2006). Use of mutually exclusive foraging areas by adjacent colonies of Blue‐Eyed Shags (*Phalacrocorax atriceps*) at South Georgia. Colonial Waterbirds, 16, 176.

